# Association of waist-to-height ratio with nonalcoholic fatty liver disease and liver fibrosis detected by vibration-controlled transient elastography: NHANES 2017-2020

**DOI:** 10.1097/MD.0000000000046675

**Published:** 2026-01-02

**Authors:** Kongting Xing, Chaoying Ge, Lingli Xu, Linjian Liu

**Affiliations:** a Longsai Hospital, Ningbo, Zhejiang, China; b Hengyang Medical School, University of South China, Hengyang, China.

**Keywords:** elastography, liver fbrosis, NAFLD, obesity, waist-to-height ratio

## Abstract

The waist-to-height ratio (WHtR) is a simple and superior indicator for assessing obesity compared with waist circumference alone. The current guidelines for nonalcoholic fatty liver disease (NAFLD), the most common chronic liver disease worldwide, do not provide any recommendations regarding WHtR. Therefore, we aimed to investigate the relationship between WHtR and NAFLD. The study analyzed the association between WHtR and NAFLD and liver fibrosis using the 2017 to 2020 National Health and Nutrition Examination Survey data. Multiple linear regression analysis and smoothed curve fitting were used to examine the relationship between WHtR and NAFLD as well as liver fibrosis. The study found a positive correlation between WHtR and hepatic and liver fat. The effect size for the correlation between WHtR and hepatic fat was (β = 18.28, 95% confidence interval: 16.16–20.41), while the effect size for the correlation between WHtR and liver fat was (β = 401.97, 95% confidence interval: 385.58–418.37). The correlation was stronger in male, non-Hispanic White participants. This study suggests a positive correlation between WHtR, NAFLD, and liver fibrosis in adults in the United States. Therefore, WHtR can be considered an important indicator for identifying the risk of NAFLD and liver fibrosis.

## 1. Introduction

Nonalcoholic fatty liver disease (NAFLD) affects 25.24% of adults,^[1]^ making it the most common chronic liver disease. In the United States alone, over 80 million people have been diagnosed with NAFLD.^[2]^ The prevalence of NAFLD has steadily increased in recent years, and its mortality rate is higher than that of the general population.^[3,4]^ NAFLD is defined as the excessive accumulation of lipids in the liver without alcohol abuse. It can progress to nonalcoholic steatohepatitis, hepatic fibrosis, and eventually hepatocellular carcinoma.^[5]^ Liver biopsy is currently the gold standard for assessing the degree of hepatic steatosis and fibrosis, but it is an invasive procedure used with caution in clinical practice.^[6]^ The liver ultrasound transient elastography (LUTE) test is a noninvasive method used to assess the degree of hepatic fibrosis and steatosis.^[7]^ The prevalence of NAFLD has increased with the rise in obesity rates.^[8–11]^ NAFLD risk is associated with increased obesity,^[12]^ and excessive fat accumulation can worsen NAFLD.^[13–15]^

In recent years, the waist-to-height ratio (WHtR) has gained significant attention due to its strong correlation with several chronic diseases, particularly obesity. WHtR is calculated by dividing the waist circumference (WC, in cm) by the height (in cm), and it serves as a straightforward screening tool.^[16]^ The WHtR is calculated by dividing the WC (in cm) by the height (in cm). WHtR and WC are highly correlated with abdominal fat as measured by imaging techniques.^[17]^ WHtR is a better predictor of early health risk in the general population than body mass index (BMI).^[18,19]^

Numerous risk factors are associated with NAFLD, and obesity is one of them. WHtR is an indicator used to monitor obesity, and it may have some diagnostic value, but its relationship with NAFLD and liver fibrosis has not been studied. We conducted a cross-sectional study to explore this relationship using data from the 2017 to 2020 National Health and Nutrition Examination Survey (NHANES).

## 2. Materials and methods

### 2.1. Subjects of study

**Figure 1.**
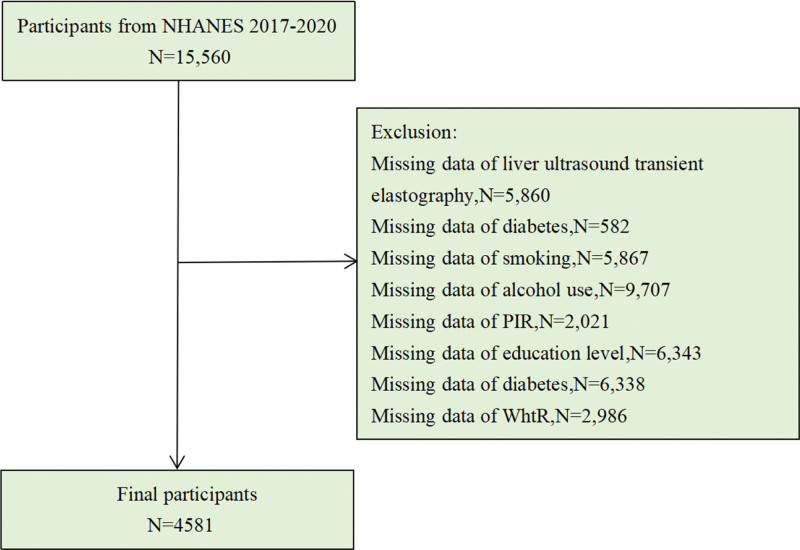
Flowchart of participant selection for the cross-sectional study.

To obtain a representative sample of the noninstitutionalized population of the United States, NHANES uses a complex multistage probability sampling strategy. Household interviews, outpatient medical examinations, and laboratory tests were used to collect data. Figure 1 displays the participant screening flowchart. Among all 15,560 subjects, we excluded missing data for each variable: LUTE (FibroScan) (n = 5680), WHtR (n = 2986), alcohol consumption (n = 9707), cigarette smoking (n = 5867), marriage (n = 6338), education (n = 6343), diabetes mellitus (n = 582), and family income poverty rate (FPIR, n = 2021). Finally, 4581 subjects were included in the analysis. The National Center for Health Statistics Research Ethics Review Board reviewed and approved the NHANES protocol. Each subject completed an informed consent form.

### 2.2. Anthropometric assessment

Height was measured using a stadiometer without shoes. The waist and hip circumference were measured to the nearest centimeter at the iliac crest and at the point of maximum protrusion of the gluteal muscles, respectively, using an elastic band. The calipers were calibrated, and the anthropometric bands were checked weekly for signs of wear according to a standardized protocol.

### 2.3. Variables

Age, gender, race (Mexican American, other Hispanic, non-Hispanic White, non-Hispanic Black, other race-including multiracial), FPIR level, education level (less than high school, high school or equivalent, college or higher), marital status (married/living with partner, widowed/divorced/separated, never married), and other demographic and lifestyle characteristics (smoking, alcohol consumption, presence of diabetes) were extracted from the household questionnaire and used as covariates. A history of diabetes was defined as a self-reported diagnosis of a specific disease. More detailed information on the above characteristics is available on the NHANES website.

### 2.4. Assessment of NAFLD and liver fibrosis

LUTE’s primary aim is to measure hepatic fibrosis and hepatic steatosis objectively. Hepatic fibrosis and hepatic steatosis are 2 important manifestations of liver disease. Liver fibrosis is measured by means of the FibroScan, which utilizes ultrasound and vibration-controlled transient elastography to derive liver stiffness. The device also simultaneously measures the ultrasound attenuation associated with hepatic steatosis and records the controlled attenuation parameter (CAP) as an indicator of liver fatness. Liver stiffness measurement (LSM) and CAP are accurate parameters that reflect hepatic fibrosis (with a sensitivity of 93.7% and specificity of 91.1%) and hepatic steatosis (with an area under the receiver operating characteristic [ROC] curve of 0.96). These parameters can be measured noninvasively using vibration-controlled transient elastography.^[20,21]^ All measurements were performed using a fiber scanner. Eddowes et al^[22]^ state that steatosis can be indicated by a CAP ≥ 274 dB/m.^[23]^ Liver fibrosis was determined using the LSM method, and according to the latest guidelines from the European Association for the Study of the Liver,^[24]^ an LSM ≥ 8.0 kPa indicates the presence of liver fibrosis.

### 2.5. Statistical analysis

This study was statistically evaluated using EmpowerStats (http://www.empowerstats.com) with a significance threshold of *P* < .05. Weighted multivariate regression models were used to investigate the association of WHtR with hepatic steatosis and liver fibrosis in 3 different models. No covariates were adjusted for in model 1. Model 2 was adjusted for sex, age, and race. Model 3 was adjusted for age, sex, race, FIPR level, education level, smoking, alcohol consumption, and presence of diabetes. Threshold effects models and smoothed curve-fitting analyses were used to verify whether there was a linear relationship between WHtR and NAFLD and liver fibrosis. In the threshold effects analysis, a log-likelihood ratio of <0.05 was used as the criterion for the existence of a linear relationship. We conducted subgroup analyses by gender, race, smoking, and the presence of diabetes, and subgroup analyses were used to assess whether the correlation between WHtR and NAFLD and liver fibrosis was stable between cohorts and to find sensitive populations.

## 3. Results

### 3.1. Characteristics of participants

Of all 15,560 individuals in the NHANES 2017 to 2020 cycle, we excluded those with missing data (Table 1). Finally, we included 4581 participants aged 20 to 80 years with complete data required for ultrasound evaluation of NAFLD, the mean (SD) age was 47.90 (16.75) years. Participants were categorized into 4 groups (Q1, Q2, Q3, and Q4) according to WHtR. The sample consisted of 51.2% males and 48.8% females, 12.05% Mexican Americans, 9.60% other Hispanics, 37.59% non-Hispanic whites, 26.11% non-Hispanic blacks, and 14.65% from other racial backgrounds. All participants’ average WHtR score was 0.60 (0.11). LSM had a mean (SD) of 5.97 (5.07) with an interquartile range of Quartile 1: 4.98 ± 2.42, Quartile 2: 5.08 ± 2.46, Quartile 3: 5.96 ± 5.45, and Quartile 4: 7.72 ± 7.31. LSM was present in 495 participants (LSM ≥ 8.0 kPa). Participants with higher LSM values were more likely to be female and non-Hispanic White than those in the quartile with the lowest LSM values. Participants with lower Married/Living with partner and FPIR were also more likely to have LSM. The mean (SD) CAP was 265.04 (62.51). Of the participants, in 2016, they had a diagnosis of fatty liver (CAP ≥ 274 dB/m). Participants with higher LSM values were more likely to be female and non-Hispanic White compared with those in the lowest LSM value quartile. Additionally, participants who married/living with a partner and had lower FPIR were also more likely to have LSM, as was CAP (Table 1).

**Table 1:** NHANES participant characteristics 2017 and 2020.

WHtR	Q1 N = 1122	Q2 N = 1030	Q3 N = 1262	Q4 N = 1167	*P*-value
Age (yr)	40.05 ± 15.85	49.01 ± 15.85	51.34 ± 16.37	50.76 ± 16.34	≤.001
Sex, n (%)					<.001
Male	605 (53.92%)	594 (57.67%)	704 (55.78%)	444 (38.05%)	
Female	517 (46.08%)	436 (42.33%)	558 (44.22%)	723 (61.95%)	
Education level, n (%)					<.001
<9th grade	20 (1.78%)	45 (4.36%)	74 (5.86%)	52 (4.48%)	
9–12th grade	94 (8.36%)	101 (9.81%)	124 (9.83%)	97 (8.31%)	
High school graduate/GED	251 (22.37%)	207 (20.10%)	274 (21.71%)	331 (28.36%)	
Some college or AA degree	371 (33.07%)	356 (34.56%)	463 (36.69%)	465 (39.85%)	
College graduate or above	386 (34.40%)	321 (31.10%)	327 (25.91%)	222 (19.02%)	
Race/ethnicity, n (%)					<.001
Mexican American	73 (6.51%)	115 (11.17%)	205 (16.24%)	159 (13.62%)	
Other Hispanic	84 (7.49%)	101 (9.81%)	149 (11.81%)	106 (9.08%)	
Non-Hispanic White	406 (36.19%)	383 (37.18%)	465 (36.85%)	468 (40.10%)	
Non-Hispanic Black	311 (27.72%)	244 (23.69%)	289 (22.90%)	352 (30.16%)	
Other race-including	248 (22.10%)	187 (18.16%)	154 (12.20%)	82 (7.03%)	
FPIR	2.78 ± 1.71	2.95 ± 1.65	2.82 ± 1.62	2.56 ± 1.57	<.001
Marital status, n (%)					<.001
Married/Living with partner	601 (53.57%)	658 (63.88%)	766 (60.70%)	670 (57.41%)	
Widowed/Divorced/Separated	162 (14.44%)	219 (21.26%)	292 (23.14%)	270 (23.14%)	
Never married	359 (32.00%)	153 (14.85%)	204 (16.16%)	227 (19.45%)	
Smoking, n (%)					<.001
Yes	466 (41.53%)	426 (41.36%)	610 (48.34%)	547 (46.87%)	
No	656 (58.64%)	604 (58.64%)	652 (51.66%)	620 (53.13%)	
Diabetes, n (%)					<.001
Yes	51 (4.55%)	118 (11.46%)	214 (16.96%)	284 (24.34%)	
No	1071 (95.45%)	912 (88.54%)	1048 (83.04%)	883 (75.66%)	
Alcohol use	2.58 ± 2.16	2.49 ± 2.09	2.54 ± 2.05	2.46 ± 2.08	.548
LSM	4.98 ± 2.42	5.08 ± 2.46	5.96 ± 5.45	7.72 ± 7.31	<.001
CAP	211.54 ± 42.56	253.08 ± 50.61	280.17 ± 52.68	310.68 ± 55.99	<.001

CAP = controlled attenuation parameter, FPIR = family income poverty rate, LSM = liver stiffness measurement.

### 3.2. Association of WHtR with liver fibrosis (LSM)

The association between WHtR and liver fibrosis was estimated. The multiple linear regression model showed a significant positive correlation between WHtR and liver fibrosis, as presented in Table 2. The study found a significant positive correlation between WHtR and liver fibrosis in all 3 models: unadjusted [11.94 (10.60, 13.28)], partially adjusted [12.43 (11.03, 13.82)], and fully adjusted [11.54 (10.12, 12.95)]. Therefore, there is a clear positive association between WHtR and liver fibrosis. Furthermore, in quartiles fully adjusted for WHtR in the model, higher WHtR was positively correlated with higher CAP and LSM in participants with Q2, Q3, and Q4 compared with those with Q1. The correlation was higher in Q4 than in Q3 and Q2 (*P* < .0001 for trend) (Table 2). These results suggest that individuals with high WHtR are more likely to develop liver fibrosis than those with low WHtR.

**Table 2:** The association between WHtR with CAP and LSM.

WHtR	Crude model	Adjusted model 1	Adjusted model 2
CAPβ (95% CI)	337.43 (323.42, 351.45) <.0001	347.08 (333.14, 361.02) <.0001	335.38 (321.30, 349.46) <.0001
WHtR group			
Q1	0	0	0
Q2	0.10 (−0.32, 0.52) .6300	−0.06 (−0.49, 0.36) .7765	−0.11 (−0.54, 0.31) .6080
Q3	0.98 (0.58, 1.38) <.0001	0.80 (0.39, 1.22) .0001	0.68 (0.27, 1.10) .0013
Q4	2.74 (2.34, 3.15) <.0001	2.76 (2.34, 3.18) <.0001	2.50 (2.07, 2.93) <.0001
*P* for trend	<.0001	<.0001	<.0001
LSMβ (95% CI)	11.94 (10.60, 13.28) <.0001	12.43 (11.03, 13.82) <.0001	11.54 (10.12, 12.95) <.0001
WHtR group			
Q1	0	0	0
Q2	41.54 (37.24, 45.84) <.0001	38.60 (34.40, 42.81) <.0001	37.46 (33.28, 41.65) <.0001
Q3	68.62 (64.54, 72.71) <.0001	65.60 (61.51, 69.70) <.0001	63.85 (59.78, 67.92) <.0001
Q4	99.14 (94.98, 103.31) <.0001	102.01 (97.82, 106.19) <.0001	98.55 (94.33, 102.76) <.0001
*P* for trend	<.0001	<.0001	<.0001

CAP = controlled attenuation parameter, CI = confidence interval, LSM = liver stiffness measurement, WHtR = waist-to-height ratio.

### 3.3. Association of WHtR with fatty liver (CAP)

The study found a significant positive association between hepatic steatosis and WHtR in multifactorial logistic regression models. However, this correlation became insignificant in the second quartile group (Q2) of WHtR in all models, including the unadjusted (*P* = .6300), partially adjusted (*P* = .7765), and fully adjusted (*P* = .6080) models. In the WHtR quartile group, participants in Q4 [2.50 (2.07, 2.93)] had higher WHtR and CAP values than those in Q3 [0.68 (0.27, 1.10)] (Table 2). These results indicate that individuals with higher WHtR are more likely to develop hepatic steatosis than those with lower WHtR.

### 3.4. Smooth curve fitting and threshold effect analysis

**Figure 2.**
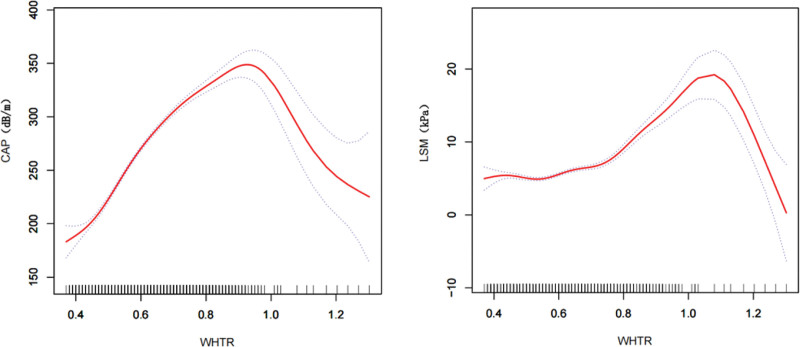
Smoothed curve fitting.

We explored whether the positive correlation between WHtR and NAFLD and liver fibrosis was nonlinear by smoothed curve-fitting analysis and verified it with a threshold effect. According to Figure 2, we found a linear relationship between WHtR and these 2 dependent variables. The results of the threshold effect model showed that when WHtR was <0.77, it was linearly associated with hepatic steatosis (log-likelihood ratio < 0.001). The positive correlation between WHtR and liver fibrosis was more significant when WHtR was >0.58, with an effect value of (OR = 18.28, 95% CI: 16.16–20.41). The results of smoothed curve fitting and threshold effect analysis are shown in Figure 2 and Table 3.

**Table 3:** Threshold effect analysis of WHtH about fatty liver and liver fibrosis.

Outcomes	CAP	LSM
Model 1, β (95% Cl) *P*-value		
Linear effect model	335.38 (321.30, 349.46) <.0001	11.54 (10.12, 12.95) <.0001
Model 2, β (95% CI) *P*-value		
Inflection point (K)	0.77	0.58
<K	401.97 (385.58, 418.37) <.0001	−2.43 (−6.02, 1.16) .1841
>K	−60.03 (−114.71, −5.34) .0315	18.28 (16.16, 20.41) <.0001
LLR	<.001	<.001

CAP = controlled attenuation parameter, CI = confidence interval, LLR = log-likelihood ratio, LSM = liver stiffness measurement.

### 3.5. Subgroup analysis of WHtR with NAFLD and liver fibrosis

We analyzed the association of WHtR with CAP and LSM in different populations and life situations using stratified weighted multiple regression analyses with subgroup analyses by gender, race, smoking, and presence of diabetes. In the subgroup analysis, we first analyzed gender. There was no correlation between WHtR and liver fibrosis in all genders, and the positive correlation between WHtR and NAFLD was stable. However, the correlation was more pronounced in male than female participants (*P* < .05). Based on the results of the study, we found that the positive correlation between WHtR and NAFLD was more significant in other Hispanic participants (*P* < .05), the positive correlation between WHtR and liver fibrosis was more significant in non-Hispanic White participants (*P* < .05). In participants with patient diabetes, the positive association between WHtR and liver fibrosis was significant (*P* < .05). Subgroup analyses of WHtR with NAFLD and liver fibrosis are shown in Tables 4 and 5, respectively.

**Table 4:** Subgroup analysis of the relationship between WHtR and NAFLD.

Characteristics	Model 1, β (95% CI)	Model 2, β (95% CI)	*P*_interaction_
Stratified by gender			<.0001
Male	441.38 (420.09, 462.67)	414.88 (393.39, 436.38)	
Female	323.79 (306.03, 341.54)	318.02 (300.21, 335.82)	
Stratified by race			.0044
Mexican American	350.55 (305.82, 395.27)	355.37 (312.30, 398.44)	
Other Hispanic	435.97 (384.11, 487.83)	423.43 (373.62, 473.24)	
Non-Hispanic White	366.70 (343.85, 389.56)	359.40 (337.07, 381.73)	
Non-Hispanic Black	317.94 (293.63, 342.26)	337.44 (313.83, 361.05)	
Other race-including	378.16 (337.01, 419.31)	366.73 (326.88, 406.58)	
Smoking			.2032
Yes	344.13 (323.16, 365.10)	353.75 (333.45, 374.04)	
No	330.05 (311.21, 348.88)	336.27 (317.68, 354.86)	
Diabetes			.5198
Yes	296.26 (257.51, 335.01)	324.06 (286.75, 361.37)	
No	327.16 (311.95, 342.38)	337.15 (322.07, 352.22)	

CI = confidence interval, NAFLD = nonalcoholic fatty liver disease, WHtR = waist-to-height ratio.

**Table 5:** Subgroup analysis of the relationship between WHtR and liver fibrosis.

Characteristics	Model 1, β (95% CI)	Model 2, β (95% CI)	*P*_interaction_
Stratified by gender			.7221
Male	14.28 (12.14, 16.43)	13.16 (10.96, 15.36)	
Female	12.71 (10.92, 14.49)	11.68 (9.86, 13.51)	
Stratified by race			.0008
Mexican American	13.18 (8.78, 17.58)	13.48 (9.09, 17.87)	
Other Hispanic	13.04 (7.94, 18.14)	11.94 (6.86, 17.01)	
Non-Hispanic White	16.31 (14.06, 18.55)	15.51 (13.23, 17.78)	
Non-Hispanic Black	8.25 (5.85, 10.64)	9.02 (6.62, 11.43)	
Other race -including	12.01 (7.96, 16.05)	10.50 (6.44, 14.56)	
Smoking			.2082
Yes	10.55 (8.54, 12.56)	11.20 (9.17, 13.24)	
No	12.90 (11.10, 14.71)	12.94 (11.07, 14.80)	
Diabetes			<.0001
Yes	22.55 (18.83, 26.27)	23.60 (19.87, 27.34)	
No	9.27 (7.81, 10.73)	9.66 (8.15, 11.17)	

CI = confidence interval, WHtR = waist-to-height ratio.

### 3.6. ROC curve of WHtR in CAP and LSM

**Figure 3.**
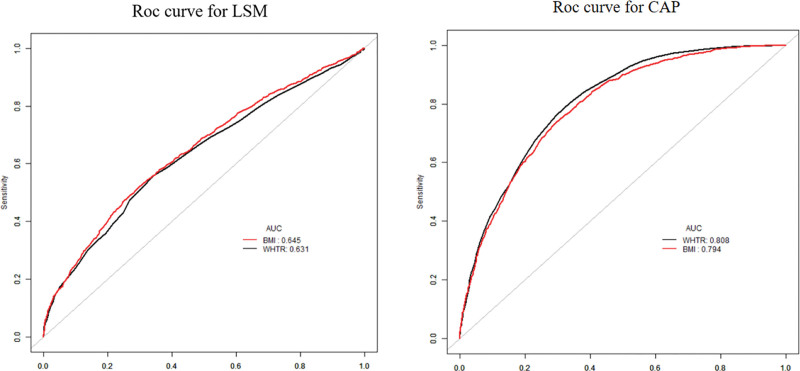
ROC curve for LSM and CAP. CAP = controlled attenuation parameter, LSM = liver stiffness measurement, ROC = receiver operating characteristic.

To better discriminate people with hepatic fibrosis from those with hepatic steatosis, we used ROC curve analysis to compare the predictive ability of WHtR in predicting hepatic fibrosis from those with hepatic steatosis.The ROC analysis showed the cutoff value (0.605), sensitivity (0.558), specificity (0.656), and accuracy (0.606) of WHtR in the assessment of hepatic fibrosis, as well as the ROC (0.630) versus the cutoff value (0.585), sensitivity (0.762), specificity (0.704), accuracy (0.733), and ROC (0.807) in hepatic steatosis. Among them, ROC analysis showed that in evaluating the relationship of WHtR over hepatic steatosis, the best predictive value of WHtR had better predictive ability in assessing hepatic fibrosis ROC (0.605) than in assessing hepatic steatosis ROC (0.585) and was statistically significant. The results of the ROC curves are shown in Figure 3 and Table 6.

**Table 6:** ROC curves for NAFLD and liver fibrosis.

Test	ROC	Best threshold	Sensitivity	Specificity	Accuracy
CAP	0.807	0.585	0.762	0.704	0.733
LSM	0.630	0.605	0.558	0.656	0.606

CAP = controlled attenuation parameter, LSM = liver stiffness measurement, NAFLD = nonalcoholic fatty liver disease, ROC = receiver operating characteristic.

## 4. Discussion

This cross-sectional study included 4581 representative participants and found a positive association between WHtR and the risk of NAFLD and liver fibrosis, which was even more pronounced for ethnicity. There is evidence to suggest that individuals with lower FPIR may be at an increased risk of developing NAFLD and liver fibrosis, as negative associations with WHtR have been observed. The results suggest that the WHtR is an important indicator for identifying the risk of NAFLD and liver fibrosis.

This study is the first to use the new WHtR to investigate the correlation between NAFLD and liver fibrosis in US adults. Recently, a more comprehensive and constructive approach has been adopted to describe NAFLD, known as MAFLD 2020. This new terminology is more appropriate and broadly based, considering the current knowledge of the increasing prevalence and etiology of NAFLD. Cai et al showed that all anthropometric indices were correlated with MAFLD. Among them, WHtR was the strongest predictor of MAFLD in young Chinese men, followed by lipid accumulation index and visceral adiposity index.^[25]^ However, our findings are limited to studies related to NAFLD.^[26,27]^ This study examined the association between WHtR and NAFLD and liver fibrosis, identifying sensitive populations in which WHtR was positively associated with the dependent variable. The first population characteristic to be validated was gender, with previous epidemiological studies demonstrating a higher prevalence of NAFLD and hepatic fibrosis in men than in women when holding other covariates constant.^[28,29]^ The prevalence of NAFLD and hepatic fibrosis has steadily increased among non-Hispanic Whites but remained stable among non-Hispanic Blacks during the 2013 to 2016 period. This trend is also observed in a study by reference ^[30]^. Staiano^[31]^ states that fat distribution characteristics differ by race, with Whites having a higher tendency to distribute fat in the viscera than Blacks. The most commonly used anthropometric measures for assessing obesity are WC and BMI. The most commonly used anthropometric measures for assessing obesity are WC and BMI. These measures are also the most important risk factors for NAFLD.^[32–34]^ A study by Pimente et al demonstrated that WHtR can be used as a valid marker of risk-associated body fat in a population of NAFLD patients. Furthermore, the researchers confirmed that the strength of the association between WHtR and whole-body and central body fat remained stable even with different WC measurement points.^[35]^ Recent studies have found that WHtR is a valid method for assessing the risk of diseases such as central obesity, type 2 diabetes, and hypertension.^[36–39]^ A further study identified WHtR as a risk factor for NAFLD and calculated the threshold and saturation effects between WHtR and NAFLD. It was determined that a WHtR of ~0.4 may be a threshold effect for NAFLD risk.^[40]^ WHtR is also considered a better indicator of the risk and severity of NAFLD and a more sensitive diagnostic tool than abdominal circumference and BMI.^[41–44]^ A study of NAFLD in children and adolescents found that when the WHtR in children and adolescents was above the threshold of 0.469, it indicated a high level of suspicion for NAFLD (sensitivity: 70.1%, specificity: 76.9%).^[45]^ Consistent with previous studies, this study found a positive association between WHtR and the risk of NAFLD and hepatic fibrosis. This suggests that individuals with higher WHtR may be more likely to develop NAFLD and hepatic fibrosis.

NAFLD is the excessive accumulation of lipids in liver cells without heavy alcohol consumption and is often accompanied by insulin resistance (IR).^[46]^ NAFLD is considered a hepatic manifestation of the metabolic syndrome and is associated with metabolic comorbidities such as obesity, hyperlipidemia, type 2 diabetes mellitus, and cardiovascular disease.^[47,48]^ The relationship between obesity and nonalcoholic fatty liver disease (NAFLD) is primarily mediated by the dysfunction of adipose tissue and hepatic de novo lipogenesis. Obesity is strongly associated with the expansion of adipose tissue, which impairs its ability to store excess energy and leads to the dysfunction of adipocytes.^[49]^ When adipose tissue dysfunction occurs, macrophages infiltrate the tissue and cause inflammation, promoting IR.^[50]^ Increasing fat cell dysfunction and IR leads to increased levels of circulating free fatty acids, which are absorbed by the liver and overwhelm its metabolic capacity.^[51]^ NAFLD is a complex condition that encompasses hepatic steatosis, nonalcoholic steatohepatitis, and cirrhosis. These pathologic changes are characterized by hepatocellular injury, fibrotic activation, and lobular necroinflammation.^[52,53]^ It is important to note that NAFLD is not a benign or static disease. Without intervention, patients with hepatic steatosis will progress to liver fibrosis over time.^[54]^ Furthermore, the analysis of the present study demonstrated that the optimal predictive value of WHtR was identified as being more effective in the assessment of the predictive ability of liver fibrosis than in the assessment of liver steatosis when evaluating the relationship between WHtR and than liver steatosis.

However, it is important to note that our study has several limitations. First, it is imaging-based and relies on fiber scans. Second, although we adjusted for many covariates, other potential confounding factors may still exist, such as physical activity and dietary intake. Finally, because this is a cross-sectional study, we cannot confirm a cause-and-effect relationship between NAFLD and the risk of liver fibrosis and WHtR. Therefore, we would like to encourage larger prospective cohort studies in the future.

## 5. Conclusion

This study found a positive association between WHtR and NAFLD and liver fibrosis in US adults. These results suggest that the WHtR may be a potential indicator for predicting NAFLD and liver fibrosis.

## Acknowledgments

We would like to thank all participants in this study.

## Author contributions

**Conceptualization:** Lingli Xu, Linjian Liu.

**Data curation:** Chaoying Ge.

**Formal analysis:** Lingli Xu, Linjian Liu.

**Funding acquisition:** Kongting Xing, Lingli Xu, Linjian Liu.

**Methodology:** Kongting Xing, Linjian Liu.

**Project administration:** Linjian Liu.

**Resources:** Lingli Xu.

**Supervision:** Chaoying Ge, Lingli Xu, Linjian Liu.

**Writing – original draft:** Chaoying Ge, Lingli Xu.

**Writing – review & editing:** Kongting Xing, Chaoying Ge.
